# COVID-19 vaccines: current and future challenges

**DOI:** 10.3389/fphar.2024.1434181

**Published:** 2024-11-06

**Authors:** Davood Mohammadi, Matin Ghasemi, Nahid Manouchehrian, Milad Zafarmand, Mitra Akbari, Amir Bahador Boroumand

**Affiliations:** ^1^ Isfahan University of Medical Sciences, Isfahan, Iran; ^2^ Islamic Azad University Tonekabon, Tonekabon, Mazandaran, Iran; ^3^ Department of Anesthesilogy, School of Medicine, Hamadan University of Medical Sciences, Hamadan, Iran; ^4^ Kurdistan University of Medical Sciences, Sanandaj, Iran; ^5^ Eye Research Center, Department of Eye, Amiralmomenin Hospital, School of Medicine, Guilan University of Medical Science, Rasht, Iran

**Keywords:** coronavirus disease (COVID)-19, COVID-19, vaccination, vaccine, immunity

## Abstract

As of December 2020, around 200 vaccine candidates for Coronavirus Disease 2019 (COVID-19) are being developed. COVID-19 vaccines have been created on a number of platforms and are still being developed. Nucleic acid (DNA, RNA) vaccines, viral vector vaccines, inactivated vaccines, protein subunit vaccines, and live attenuated vaccines are among the COVID-19 vaccine modalities. At this time, at least 52 candidate vaccines are being studied. Spike protein is the primary protein that COVID-19 vaccines are targeting. Therefore, it is critical to determine whether immunizations provide complete or fractional protection, whether this varies with age, whether vaccinated people are protected from reoccurring diseases, and whether they need booster shots if they’ve already been inoculated. Despite the enormous achievement of bringing several vaccine candidates to market in less than a year, acquiring herd immunity at the national level and much more so at the global level remains a major challenge. Therefore, we gathered information on the mechanism of action of presently available COVID-19 vaccines in this review and essential data on the vaccines’ advantages and downsides and their future possibilities.

## 1 Introduction

The severe acute respiratory syndrome coronavirus 2 (SARS-CoV-2) virus first appeared at the end of 2019. Fever, cough, dyspnea, malaise, fatigue, and sputum/secretion are some of the clinical signs of Coronavirus Disease 2019 (COVID-19), which is caused by SARS-CoV-2 infection. It quickly spread worldwide, prompting the World Health Organization (WHO) to declare a worldwide pandemic on 11 March 2020. This novel *Betacoronavirus* is related to the coronaviruses that cause severe acute respiratory syndrome (SARS) and Middle East respiratory syndrome (MERS) (Hu et al., 2021). Other common symptoms include neurological symptoms, dermatological manifestations, anorexia, myalgia, sneezing, sore throat, rhinitis, goosebumps, headache, chest pain, and diarrhea (da Rosa Mesquita et al., 2021). Reducing COVID-19 spread requires efficient treatments, and vaccinations are regarded as a standard and effective approach for controlling infectious illnesses (Hajj Hussein et al., 2015; Tregoning et al., 2021). Regarding the challenges scientists and vaccine developers face during designing proper vaccines against this novel coronavirus, it is important to have a comprehensive overview on the history of vaccination against viral infections as well as mechanism of action of SARS-CoV-2 from direct pathogenesis to immune-related one which all are considered in this review.

Over 200 vaccine candidates for COVID-19 have been developed as of December 2020 and at least 52 potential vaccines have been tested in humans. And by December 2021, several other vaccines were moved from Phase I/II to Phase III. The United Kingdom (United Kingdom) was the first country to approve the COVID-19 vaccine, BNT162, developed by Pfizer and BioNTech, through Emergency Use Authorization (EUA) on 2 December 2020. On 31 December 2020, the WHO approved BNT162 for emergency usage, making worldwide manufacturing and distribution easier. Several governments, including the United States, Canada, Russia, China, and India, have used similar EUA processes to approve various COVID-19 vaccine candidates (CVCs), and the list keeps growing (Kashte et al., 2021). COVID-19 vaccines have been and are being developed on a variety of platforms. Some are established techniques, such as an inactivated virus or live attenuated viruses, which have been employed in inactivated influenza vaccines and measles vaccinations, respectively. Newer platforms, such as recombinant proteins (used in human papillomavirus vaccines) and vectors (used for Ebola vaccines), are used in other techniques. RNA and DNA vaccines, for example, had never been used in an approved vaccine (Krammer, 2020). This review primarily gathered information on the mechanism of action of vaccines against viral infections and particularly currently available COVID-19 vaccines; we also offered critical data on the benefits and drawbacks of these vaccines and their prospects. Four databases including Scopus, Web of Sciences, Embase and PubMed were searched with the following keywords, “severe acute respiratory syndrome coronavirus 2”, “SARS-CoV-2”, “Coronavirus Disease 2019”, “COVID-19”, “vaccine”, “vaccination”.

## 2 Vaccines: An overview

The commencement of the current vaccination era is related to the English physician Edward Jenner, who is credited with one of the utmost considerable findings in public health, dating back to 1796. Before Jenner, however, a procedure known as ‘inoculation’ or ‘engrafting’ was commonly used in Asia Minor and the Far East (Lombard et al., 2007). Jenner was not the first scientist to uncover that people who had cowpox did not get smallpox; however, he was the first to show that cowpox pus from an infected person could be used to immunize another person, resulting in the first vaccine (Willis, 1997). Many experts throughout the world have focused their study on vaccination as a result of this discovery, supporting immunological improvements. Smallpox, as a potentially fatal infections in the history of humankind, was declared eradicated by WHO in 1980 as a consequence of global vaccination programs, and many infectious diseases that were once assumed serious public health threats, such as measles, polio, diphtheria, and whooping cough, are no longer a priority in most parts of the world, ows to decades of worldwide immunization efforts (Tang et al., 1992). Vaccines work by triggering an innate immune response, generating an antigen-specific adaptive immunological response, similar to how natural infections do. The initial line of defense against infections that have infected the body is innate immunity. It takes only a few hours to establish, but it is not specific to any pathogen and has no memory (Clem, 2011). Adaptive immunity is the second line of defense that emerges later in the infection process and is defined by a large number of lymphocytes and antibodies that can identify and eradicate nearly any pathogen. Each pathogen (or vaccine) expresses (or includes) antigens that stimulate B lymphocytes to generate specific antibodies and activate cell-mediated immunity by stimulating extremely specific subsets of T cells (Seder and Hill, 2000). The adaptive immune system establishes immunological memory after the infection is eradicated. The persistence of antibodies and the production of memory cells, which can quickly reactivate upon subsequent exposure to the same pathogen, constitute immunological memory, which is the foundation of long-term protection and the purpose of vaccination (Pulendran et al., 2013). Although vaccines are generally seen as strategies for individual protection, they can also safeguard unprotected populations by lowering the incidence of person-to-person infection and the chance of individuals becoming infected. This indirect protection, known as herd or community protection, necessitates the vaccination of a large section of the population (75–95 percent depending on the disease) or a special group that plays a central role in disease transmission. Herd protection is frequently required for vaccination programs to be successful, such as those for measles (Rashid et al., 2012).

Historically, producing an effective and safe vaccination took 10 years, beginning with early preclinical investigations, Phase I-III human clinical trials, and finally, the arduous approval process by national and international regulatory authorities (Pronker et al., 2013). Phase I includes considering the effectiveness and basic safety of the vaccine candidate in a minor population (20-200) of healthy people. Phase II includes a bigger sample group of several hundred people to gather more data on the vaccine candidate’s safety, immunogenicity, efficacy, and optimum dose. Finally, phase III covers thousands of people and examines the vaccine candidate’s safety and efficacy in bigger populations and identifies real-world reactions and evaluates usefulness by comparing vaccinated and unvaccinated categories (Plotkin et al., 2017). If these data combined show appropriate safety and effectiveness, companies can present authorization applications to different regulatory countries (Eroglu et al., 2021).

To comprehend the benefits and hazards of vaccines, it is necessary to understand the basic ideas of vaccines and the guidelines for their usage. The number of cases and deaths from infectious illnesses stopped worldwide due to direct and indirect effects of vaccination, as well as the economic benefit to society from the cost of prevention, should be recognized. Improving public awareness about vaccinations might assist minimize vaccine hesitation by supporting reasonable vaccine expectations and increasing confidence in vaccine research.

## 3 Natural and vaccine-induced immunity

Vaccination’s central concept is the proactive stimulation of a protective immune response by imitating an infectious pathogen’s (bacteria, viruses, *etc.*) natural interaction with the human immune system (Toor et al., 2021). During an encounter with an infectious agent, the immune system devises and refines a defense plan that stops the pathogen from spreading further, disrupts its life cycle, and ultimately removes it from the body. Following that, the individual patient should develop protective immunity, which will prevent subsequent infections by the same agent (Nicholson et al., 2016). As with any immune system response, the body must first perceive the threat as a pathogenic agent or an immunization. The innate immune system normally carries out the initial recognition, while B-cells may also be implicated. The recognition phase begins whenever the immune system identifies epitopes on antigens. Antigen epitopes are tiny subregions that mimic immunological responses. The innate immune system will then react in a number of ways to the stimulation. The antigen-presenting cells (APCs), such as macrophages or monocytes, will opsonize or bind to the agent, helping them swallow the infectious agent (Xagorari and Chlichlia, 2008). These APCs will then digest the pathogenic agent’s antigens and insert them into the APCs’ surface, along with the MHC protein. If the antigen is a viral antigen, the APC will bind it to MHC I protein and present it to a CD8 cell, eliciting cell-mediated immunity. If the antigen is a bacterial or parasite antigen, the APC will attach it to MHC II protein and deliver it to a CD4 cell, causing an antibody-mediated reaction. Immunization is defined as the intentional promotion of an adaptive immune response. (Davis and Gack, 2015). Immunization, also known as vaccination or inoculation, promotes resistance in the human body to particular diseases by employing modified or destroyed microbes (bacteria or viruses). These treated microorganisms do not cause infection; they stimulate the immune system to develop a protective mechanism that protects the body from the infection (Levine and Sztein, 2004). When a person who has been immunized against a disease comes into touch with the disease-causing agent, the immune system is able to respond defensively almost instantly. Immunization can be obtained in two ways: passive or active. These resources might come from both natural and artificial sources. Exposure to the environment, humans, and animals are all natural sources. On the other hand, artificial sources result from medical procedures (Wilby and Werry, 2012).

## 4 SARS-CoV-2 virology and vaccination

CoVs derive their name from their crown-like appearance under an electron microscope (EM), which results from the surface glycoproteins on the virus. They are enveloped in positive-sense single-stranded RNA viruses that can infect birds and animals and cause a variety of respiratory, gastrointestinal, hepatic, renal, and neurologic illnesses (AbdulRahman et al., 2024). Presently, circulating CoVs in humans comprise two α-CoVs (229E and NL63) and two β-CoVs (OC43 and HKU1) that cause the common cold. SARS-CoV-1, the Middle Eastern respiratory syndrome coronavirus (MERS-CoV), and the more recent SARS-CoV-2 are examples of extremely pathogenic human β-CoVs that have developed in the last 2 decades (Cui et al., 2019). Adopting a ribosomal frameshifting strategy, the SARS-CoV-2 genomic RNA comprises a 5′cap and a 3′poly(A) tail that allows rapid translation to form two coterminal replicase polyproteins, pp1a and pp1ab. Two-thirds of the genome is encoded for replicase polyproteins (ORF1a and ORF1b). These polyproteins are then cleaved into individual non-structural proteins (nsps), nsp1-11 and nsp1-16, by two viral proteases (nsp3-PLpro and nsp5-Mpro), respectively (Fehr and Perlman, 2015). The remaining third is responsible for structural (spike (S), envelope (E), membrane (M), nucleoprotein (N) and accessory proteins (ORF-3a, -3b, −6, -7a, -7b, −8, -9a, -9b, and 10) (Zhou et al., 2020). As with SARS, there is currently no licensed vaccination for MERS (Folegatti et al., 2020). The failure of SARS and MERS vaccines can be attributed to many factors. Due to a lack of functional and cost-effective small animal models, vaccine development for MERS was impeded in the pre-clinical stages. Furthermore, because MERS has been intermittent and geographically limited, less emphasis has been dedicated to developing MERS vaccines. When it comes to SARS, it's impossible to invest in vaccine development because no disease cases have been documented since 2014, implying that SARS-CoV has vanished (Padron-Regalado, 2020). The central protein considered a target in COVID-19 vaccines is the S protein. S protein is a homotrimer composed of a membrane-distal S1 subunit and a membrane-proximal S2 subunit that resides in the virus envelope. The S1 subunit, through its receptor-binding domain (RBD), regulates receptor recognition, while the S2 subunit is in charge of membrane fusion, as an essential factor for virus entry. Neutralizing antibodies (nAbs) can hypothetically attack the S protein to block viral infection at many points during infection’s beginning phases. The RBD is the most common target of nAbs that interact with viral specific receptors. The majority of successful nAbs against SARS-CoV-2 have so far targeted the RBD. nAbs attacking the N-terminal domain (NTD) have also been discovered in SARS-CoV-2 and MERS-CoV infections, which indicates that it may be even included in the vaccine. The S2 subunit might be targeted by nAbs that interact with the structural redisposition of the S protein and the insertion of fusion peptide (FP) compulsory for virus-host membrane fusion (Dai and Gao, 2021). The vaccines from Pfizer/BioNTech and Moderna are RNA-based, whereas the vaccination from Oxford-AstraZeneca uses a non-replicating viral vector technology to deliver SARS-CoV-2 DNA. Most potential vaccines in current trials target spike protein subunits; however, escape mutations are a real possibility. Spike variants that evade vaccine targeting are referred to as escape mutations. Mutations in this protein subunits allowing the virus to escape the immune response and potentially reduce the vaccine’s effectiveness. When antibodies bind to a virus but do not neutralize it, the Fc-region of the antibody binds with Fc-receptors on immune cells such as macrophages, causing viral uptake. After that, the virus can increase within the host cells, resulting in immunopathology. Using a live-attenuated form of the complete virus (measles, mumps, rubella, and others) or an inactivated virus (hepatitis A, rabies, and others) to generate a polyclonal response has been one strategy to discourage or circumvent escape strains. In reality, China’s Sinovac vaccine, which is presently in Phase 4 testing, uses inactivated virus. Nevertheless, even in the presence of polyclonal antibodies, SARS-CoV-2 can evade neutralization, as evidenced by convalescent plasma therapy from COVID-19 survived individuals (Malik et al., 2021). Although no vaccine targeting nucleocapsid protein has started clinical trials, ImmunityBio, Inc. and NantKwest Inc.'s vaccine candidate, a Human Adenovirus Type 5 Vector (hAd5) expressing Spike (S) + Nucleocapsid (N), has reached a phase I of a human clinical study. Coronavirus M protein plays a significant role in virus assembling (Neuman et al., 2011). M proteins, along with minor levels of E, are located amid the S proteins in the viral envelope and are essential for virus budding. OncoGen, one of the SARS-CoV-2 vaccine manufacturers, has proposed a synthetic long peptide vaccine candidate that targets SARS-S CoV-2’s and M proteins; yet, pre-clinical testing results have to be released. Inactivated and live-attenuated vaccines are designed to target the whole virus. They include all structural proteins (S, N, M, and E proteins) and non-structural and auxiliary proteins, which can be produced *in vivo* by live-attenuated viral vaccines. As a result, such vaccine (like PiCoVacc, developed by Sinovac Biotech or sCPD vaccine) candidates can induce bigger antibody and T cell responses than the previous section vaccines, which are based on a single protein or protein fragments.

## 5 Technologies used for COVID-19 vaccine development

**FIGURE 1:**
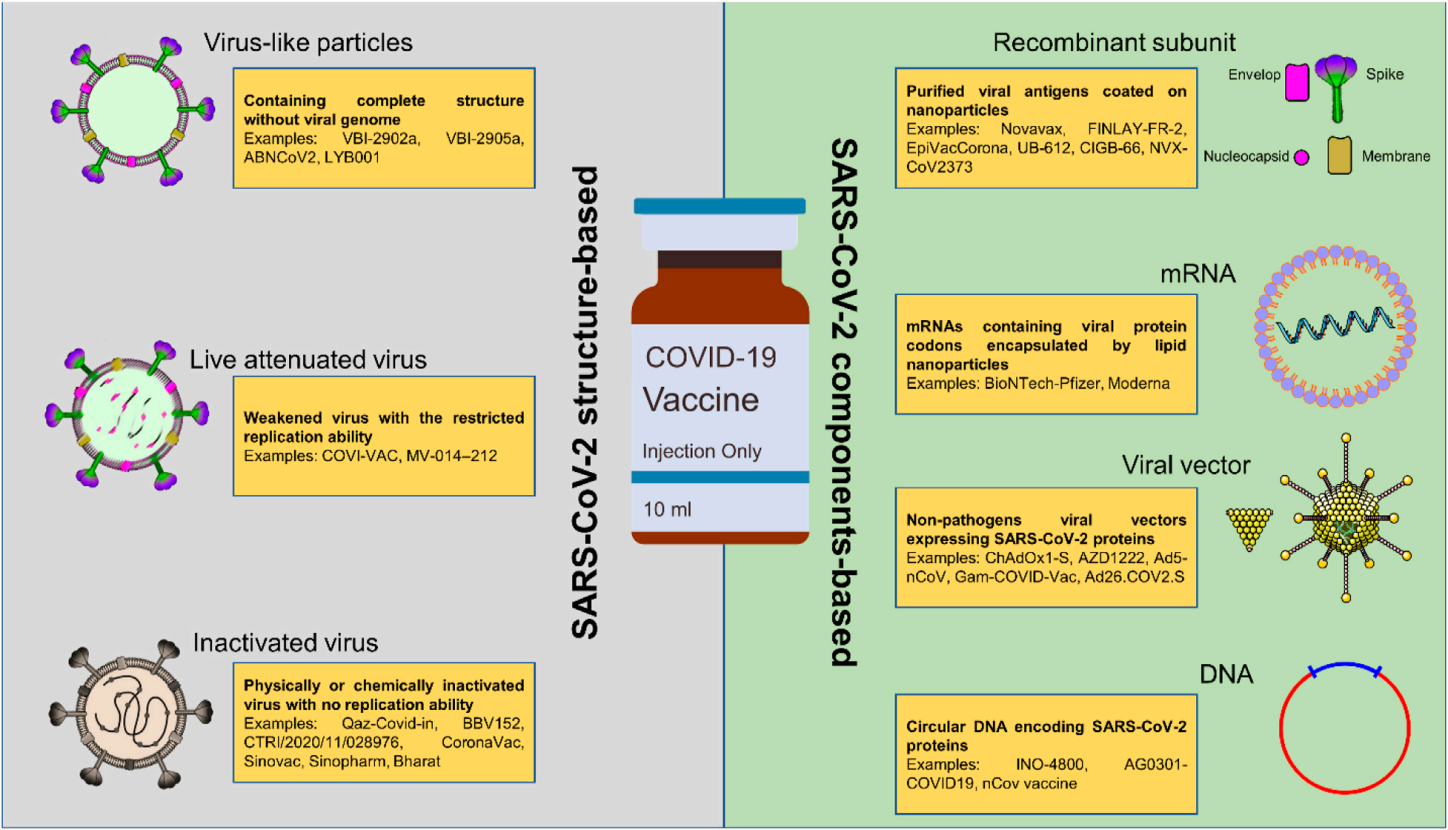
The currently under consideration platforms used in producing COVID-19 vaccines. Most of these platforms are based on the components of SARS-CoV-2; however, the structure-based COVID-19 vaccines are easier to access and manufacture due to their similarity to previously approved viral vaccines.

The prolonged pandemic caused by COVID-19 has presented researchers working on vaccines with both opportunities and problems. Researchers working on vaccines, public health agencies, and political leaders from all around the world realized from the very beginning that the most effective method to battle the epidemic would be to produce immunizations that really work. Unlike to the case with influenza vaccinations, there were no coronavirus vaccines on the market before the COVID-19 pandemic. There has been a concerted effort made to drastically reduce the amount of time needed to create a new vaccine in response to the pressing and widespread need for a completely new COVID-19 vaccine (Han, 2015). In order to be prepared for the COVID-19 pandemic in a timely manner, a number of potential COVID-19 vaccines have been investigated using a wide range of technologies and research platforms (Figure 1). These COVID-19 vaccine candidates include nucleic acid (DNA, RNA) vaccines, viral vector vaccines, inactivated vaccines, protein subunit vaccines, and live attenuated vaccines (Amanat and Krammer, 2020; Lurie et al., 2020).

Plasmids are used to produce DNA vaccines, which involve inserting the pathogen’s antigen-encoding DNA into the plasmid (antigenic components of SARS-CoV-2 such as spike protein). These are not known to pose any health risks and are not capable of causing disease. However, there is a lack of clinical scientific proof for these types of vaccinations. When taken by themselves, they have the potential to cause adverse events (ADE) (Dai et al., 2019; Khuroo et al., 2020). These vaccines are highly immunogenic; when given in conjunction with inactivated vaccines, they produce a high titer of antibodies capable of neutralizing the disease (Khalid and Poh, 2023). In order to properly deliver these vaccinations, electroporation is necessary. At the moment, there are fourteen candidates for the COVID-19 vaccine that are conducting preclinical testing on the DNA platform, in addition to the four candidates that are now undergoing clinical testing. One example of a vaccine that has been designed to fight COVID-19 is called INO-4800, and it was developed by INOVIO Pharma, the Korean Institute of Health, and the International Vaccination Institute (Kaur and Gupta, 2020).

Virus vector vaccines are made using recombinant DNA technology. The pathogen’s antigen-coding DNA is introduced into the bacteria or virus vectors. The antigen is then expressed in these cells by these bacteria or virus vectors. The antigens are extracted from the bacterium or virus vectors and purified. Vaccines based on viral vectors can be replicating or nonreplicating. A virus unrelated to the one in question, such as measles or adenovirus, is genetically modified to encode the gene of interest. These are thought to be safe and capable of eliciting a significant T and B cell response. Hepatitis B, HPV, and pertussis vaccines are some of these vaccines (Kaur and Gupta, 2020). These vaccines have not yet been approved by the FDA (Dai et al., 2019). Ad5-nCoV, developed by CanSino Biological Inc./Beijing Institute of Biotechnology, and ChAdOx-nCoV-19, developed by the University of Oxford, are two examples of COVID-19 vaccines. Also, Ad26.COV2.S from Johnson and Johnson is a recombinant, non-replicating adenovirus serotype 26 (Ad26) vector carrying the full-length and stable SARS-CoV2 spike protein. Ad5 vector from CanSino’s COVID-19 vaccine, based on Wuhan Hu1 and encoding a full-length spike gene, is replication-deficient (Ling et al., 2021).

RNA vaccines in the form of mRNA require an additional step between the replication of DNA and the ribosome-mediated translation of proteins. Both non-replicating mRNA and RNA that amplifies themselves on their own are types of mRNA vaccines that are now the subject of investigation. The protein of interest is encoded by non-replicating mRNA that contains between 50 and 30 untranslated regions (UTRs). Still, the self-amplifying RNA encodes the protein/antigen and the viral replication machinery, which allows for intracellular RNA amplification and enormous levels of protein production (Fuller and Berglund, 2020). Even though they both function comparably, it would appear that immunizations based on RNA are more effective and safer than those based on DNA. Because RNA does not need to enter the nucleus of the cell in order to function properly, its injection into cells poses no danger, or only very low risk, of altering the original DNA sequences (Dolgin, 2021). In the fight against SARS-CoV-2, mRNA vaccines have emerged as one of the most promising potential intervention. mRNA vaccines are BNT162b2 (Pfizer/BioNTech, New York, NY, United States/Berlin, Germany) and mRNA-1273 (Moderna, Cambridge, MA, United States), have received both approved for use in emergencies around the world. The BNT162b2 vaccination received full FDA clearance not too long ago. In systematic review study on Pfizer vaccine it was demonstrated that The average number of side effects across 14 studies was 77.34% for injection site pain, 43% for fatigue, 39.67% for muscle pain, 33.57% for local swelling, 33.27% for headaches, 25.75% for joint pain, 18.34% for chills, 18% for fever, 9.38% for itching, 7.86% for swelling of the lymph nodes, 7.86% for nausea, 7.86% for dyspnea, and 6.36% for diarrhea (Dighriri et al., 2022).

The earliest vaccines used were live attenuated vaccines. These vaccines are made by cultivating bacteria in less-than-ideal circumstances or passing them through cultures multiple times, approaches that determine pathogenicity attenuation while keeping the ability to generate an immune response (Lauring et al., 2010). BCG, Smallpox, and Polio vaccinations are examples of live attenuated vaccines. DelNS1-SARS-CoV2-RBD, developed by the University of Hong Kong, is an example of a COVID-19 vaccine (Khuroo et al., 2020).

Heat, radiation, or chemicals such as formaldehyde or β-propiolactone are used to produce inactivated vaccines. This is done in order to partially destroy viral structure and/or the genetic material (Sabbaghi et al., 2019). These immunizations still include all viral components; however, they are in an inactive condition that prevents them from producing illness in humans. Inactivated vaccines are often thought of as being safe, easy to develop and produce, and less immunogenic. Consequently, they may not elicit a significant immune response, which necessitates the use of adjuvants and/or several doses of the vaccine (Sanders et al., 2015). Inactivated vaccines are used to prevent diseases such as hepatitis A and rabies, for example,. One such vaccine, called PiCoVacc, was developed by Sinovac Biotech and is intended to protect against COVID-19 (Kashte et al., 2021).

To elicit powerful immune responses, subunit vaccinations are usually made of non-genetic viral proteins or peptide fragments. Subunit vaccinations are safer since they don't contain a fully infectious virus, which removes the risk of virus inactivation or toxicity reversal. For example, most SARS-CoV-2 subunit vaccines target proteins, mainly the spike protein or age proteins in specific locations, according to studies; however, some subunit vaccines target N proteins (Yan et al., 2021). NVX-CoV2373, a vaccine developed by Novavax, is one example of such a vaccine to combat COVID-19 (Khuroo et al., 2020).

Virus-like particles (VLPs), spontaneously generated from viral structural proteins, have increasingly emerged as vaccine delivery agents (Yuan et al., 2020). These are multimeric structures that can directly trigger immune cells by replicating the three-dimensional shape of natural viruses. Furthermore, VLPs are free of infectious, genetic material, unlike attenuated or inactivated virus products, making them intrinsically safer (Chen and Lai, 2013; Dhawan et al., 2023). They include functional viral proteins that enable the virus to penetrate cells, allowing for efficient cell entrance (and consequently tissue-specific targeting specified by the virus’s origin), making them a potential candidate for vaccine development. Two COVID-19 vaccine candidates created as VLPs are now in clinical trials, while 15 COVID-19 vaccine candidates are in the preclinical stage of development. Premas Biotech’s Triple-Antigen Vaccine is one example of a COVID-19 vaccine (Khuroo et al., 2020).

## 6 Immunity generated by COVID-19 vaccines

The immunological response to SARS-CoV-2 is influenced by innate immune activation and antigen-specific responses of B and T cells (Thevarajan et al., 2020). The bulk of protection against viral infection comes from virus-neutralizing antibodies, which is true for the wide range of viral infections against which humans develop considerable immune protection through infection or vaccination. Therefore, vaccines that generate protective immune responses, such as virus-neutralizing antibodies specific to SARS-CoV-2, must be developed soon (Gates, 2020). Virus-neutralizing antibodies are the primary mechanism of protection elicited by currently available vaccinations. Antibodies of these kinds more often prevent the virus from engaging with its cellular receptor or prevent the virus from going through the conformational changes that are necessary for the virus to fuse with the cell membrane (Tada et al., 2021). The most apparent isotype produced by a COVID-19 vaccine is IgG, particularly the more protective IgG1 and IgG3 subtypes (Klingler et al., 2021; Espino et al., 2024).

On the other hand, IgA may play a significant role in the prevention of infection of mucosa and epithelial cells in the respiratory system, in addition to endothelium cells, which the virus may target broadly. While mass mucosal immunization in a timely manner may be problematic, introducing an adjuvant that stimulates the formation of IgA may be an essential factor. TLR7/8 and TLR9 ligands are promising possibilities since they stimulate IgA responses effectively (Bessa and Bachmann, 2010; Meiler et al., 2008). SARS-CoV-2 antigens are recognized and reacted to by CD4 and CD8 T cells, which contribute to immune defense by lowering disease severity (Ahmed et al., 2020; Sridhar et al., 2013). However, when it comes to disease prevention, T cells on their own are probably not as efficient as antibodies that can neutralize pathogens. CD4 T helper cells are critically crucial for both the responses of B cells and the production of antibodies.

As a consequence of this, vaccinations have to excite B cells in addition to T cells simultaneously (Yang et al., 2004). In clinical trials, the first vaccines that utilized SARS-CoV-2 S proteins by mRNA lipoparticles (Pfizer BNT162b2; Moderna VRC mRNA) or viral vectored vaccines (CanSino AdV5 COVID-19; Oxford/AstraZeneca ChAdOx) indicated high efficiency for SARS-CoV-2-specific CD4^+^ and CD8^+^ T cells. This efficiency was evaluated by IFN-γ release and ELISpot assays (Sahin et al., 2021; Zhu et al., 2020). After immunization, there was a 10-fold increase in IFN-γ secreting T cells compared to baseline, which was comparable to the amount of IFN-γ producing T cells seen in COVID-19 patients. Furthermore, after the COVID-19 vaccination, IFN-γ+CD4^+^ T cells numerically dominated over IFN-+CD8^+^ T cells, similar to what was observed after SARS-CoV-2 infection and COVID-19 vaccination in non-human primates (Krammer, 2020). While most of the currently licensed COVID-19 vaccines provide protection against moderate to severe disease with just one dosage, most SARS-CoV-2 vaccinations require two doses to provide full and long-lasting protection (Garcia-Beltran et al., 2021).

## 7 Preclinical evaluation of vaccine efficacies and safety

According to research on the adenovirus vector vaccine, around 1,010–1,011 virus particles successfully encouraged the generation of neutralizing antibodies against RBDs during 0–28 days following immunization, which occurred somewhere between days 0 and 28. Furthermore, research using mRNA vaccines indicated that 25–30 μg dosages might cause neutralizing antibody responses in people within 0–28 days of vaccination. After 57 days, people who had received two doses of the subunit vaccine for the purpose of one Australian study had antibodies that were neutralizing against the virus. These individuals numbered 67 out of 68. Within 28 days of vaccination, vaccinated individuals were able to elicit anti-S IgG and neutralization responses when they were given the recombinant S protein nanoparticle vaccine (NVX-CoV2373), according to the findings of another research (Pormohammad et al., 2021). The Food and Drug Administration (FDA) has provided a paper containing recommendations to the pharmaceutical sector about producing COVID-19 vaccines (Food and Administration, 2020). “The general safety evaluation of COVID-19 vaccines, including the size of the safety database to support vaccine licensure, should be no different than for other preventive vaccinations for infectious illnesses,” the FDA notes in the document. Two phase III trials have recently started enrolling patients in accordance with the recommendations. Each study has a target of 30,000 participants, a quantity that reflects the fact that there have been no previously approved COVID-19 vaccines to assess the effectiveness and safety (gov, 2020). According to recent research, the T cell and antibody responses generated by a single dose of the BNT162b2 vaccine were equal to those naturally infected with SARS-CoV-2 during weeks or months following infection (Angyal et al., 2022). In certain nations, the administration of the second dosage was delayed for up to 12 weeks so that the initial dose may be administered to a greater number of people (Pimenta et al., 2021). For instance, Canada had reached an agreement to delay administering the second dose for 16 weeks (Tauh et al., 2021).

However, due to an inadequate immune response in those who only got a single dose of the vaccine, such a technique can potentially contribute to the creation of SARS-CoV-2 variants (Kadire et al., 2021). In a recent investigation, neutralizing antibody titers during the peri-infection phase were found to estimate the likelihood of breakthrough SARS-CoV-2 infections in fully vaccinated populations (Bergwerk et al., 2021). The significantly mutated changes were found to be indicative of a form of fast, multistage evolutionary leaps that might particularly occur in the situation of poor immune control (Truong et al., 2021; Choi et al., 2020). In order to prevent extended SARS-CoV-2 infections, which might lead to the establishment of multimutational SARS-CoV-2 variants, immunocompromised individuals should be given priority for anti-COVID-19 immunization (Corey et al., 2021). According to the findings of several studies, the third dosage of Moderna, Pfizer-BioNTech, Oxford-AstraZeneca, and Sinovac generated a significant increase in the number of infection-blocking neutralizing antibodies when it was given a few months after the administration of the second dose (Hause et al., 2021; Flaxman et al., 2021; Peled et al., 2022). Furthermore, the common side effects of the third dose, which ranged from mild to moderate, were not substantially different from the symptoms associated with the first two doses (Hause et al., 2021). Following administration of the third dose of the BNT162b2 vaccine, the cumulative incidence rate of both local and systemic adverse effects was 69 percent (57/97), with the latter accounting for 20 percent (19/97) of the total (Peled et al., 2022). According to the observations of the phase III clinical trial that included 306 individuals aged 18–55 years, adverse effects that were disclosed after receiving a second dose of the BNT162b2 vaccination were parallel to those that were noted after receiving a third dose of the vaccination 5–8 months after the completion of two doses (Hause et al., 2021).

## 8 Challenges and opportunities in COVID-19 vaccines (advantages and disadvantages)

It is of the utmost importance to determine if vaccinations offer complete or only partial protection, if this fluctuates with age, whether vaccinated persons are protected from recurring infections, and whether they require booster injections if they have already been immunized (which would demand the manufacturing of billions of extra doses) (Table 1). According to prior animal testing with vaccinations against similar coronaviruses that cause SARS and MERS, weak antibody levels might lead to aberrant immune responses (Shen et al., 2022; Merad et al., 2021). Antibodies directed against the SARS-CoV S proteins may have a part to play in the phenomenon known as antibody-dependent enhancement (ADE), which refers to an intensification of infection (Iwasaki and Yang, 2020). Additionally, cell-based enhancement is likely, which may involve allergic inflammation caused by Th2 immunopathology following vaccination (Tseng et al., 2012). To this day, sixteen inactivated SARS-CoV-2 vaccines have been developed and are now undergoing testing in clinical studies (Organization, 2020). Inactivated vaccines often have acceptable efficacy and safety. Compared to other vaccines, they have a relatively short production cycle. However, the vaccine’s efficacy against mutated strains is partially compromised, requiring additional booster doses (Chen et al., 2021; Vacharathit et al., 2021; Zhang et al., 2022a). Concerns remain regarding the use of inactivated virus vaccine platforms against COVID-19, despite the impressive success of these vaccines. Some of these concerns relate to the difficulty of verifying complete virus inactivation status, which is a risk that could ultimately lead to a situation that is comparable to the Cutter incident of 1955, in which children who received the polio vaccine were infected with the inactivated poliovirus (Organization, 2020; Nathanson and Langmuir, 1963). Vaccinated animals still display severe disease when challenged, despite the fact that various established inactivated SARS-CoV vaccines have been demonstrated to elicit nAbs. This might explain why there are presently no vaccinations for SARS permitted to be used in the United States (Roper and Rehm, 2009). The attenuated virus in live attenuated vaccines has the potential to multiply and propagate within the host, which results in cheaper manufacturing and delivery costs. This is one of the many benefits that live attenuated vaccines have over their inactivated counterparts. As a consequence of this, a low dosage of the virus may be all that is necessary to induce immunity (Mak et al., 2006). Additionally, live attenuated vaccines may be given intranasally, which allows the attenuated virus to increase in the mucosal tissue of the upper respiratory tract. This is important since the upper respiratory tract is the major entry site for coronaviruses (Tiboni et al., 2021). Currently, there are only six SARS-CoV-2 live attenuated virus vaccines that have been developed, four of which are in the pre-clinical phase and two of which are in phase I clinical trials (Organization, 2020). Despite the benefits and accomplishments of employing live attenuated viral vaccines to battle numerous infectious illnesses, the overt danger of using such a vaccine remains the use of a live replicating virus, which can revert to its pathogenic phenotype under any environment, causing sickness after immunization, especially in immunocompromised people (Dong et al., 2020). One such preventative measure is using a vaccine against infectious agents derived from viral vectors. These vaccines are incredibly accurate in delivering genes to the cells that need them, efficient in moving genes from one place to another, and successful in terms of provoking an immune response (Ura et al., 2014). They have a long-term and high degree of antigenic protein expression; hence, they promise preventive utilization because these vaccines trigger and boost cytotoxic T lymphocytes (CTL), which subsequently target virus-infected cells (Andreadakis et al., 2020). In order to create DNA vaccines, it is necessary to clone the SARS-CoV-2 S gene onto bacterial plasmids with a strong mammalian promoter, such as CMV and/or SV40, and to generate enormous amounts of plasmids in bacteria that are capable of doing so. As the first proof-of-concept DNA vaccine, it was developed in 1990 by injecting DNA vectors into the skeletal muscle of mice. These vectors expressed chloramphenicol acetyltransferase, luciferase, and beta-galactosidase (Wolff et al., 1990). Plasmid DNA vaccines offer several advantages, including the capability to target and drive both humoral and cellular immune responses; flexible and easy large-scale production and formulation methods across short timelines, which makes them suitable for COVID-19 crisis response; capacity for multivalency; and the ability to be held the finished vaccine at room temperature. However, there are some significant disadvantages to using this sort of vaccine: (1) Low immunogenicity in humans, necessitating multiple vaccine doses to acquire optimal protection. (2) Carcinogenesis risk due to possible cellular chromosome integration (Frederiksen et al., 2020). At the beginning of the pandemic, BioNTech and Pfizer had intended to develop five different COVID-19 mRNA vaccine candidates based on nucleoside-modified mRNA (BNT162b2, BNT162b1, and BNT162b3), non-modified mRNA (BNT162a1), and self-amplifying mRNA (BNT162a1) (BNT162c2). To this point, all of these potential candidates have been tested in clinical settings (Fang et al., 2022). The safety, speed, and design and production flexibility of mRNA vaccines are key advantages (Pardi et al., 2018; Zhang et al., 2022b). The mentioned vaccines are easy to design and manufacture because neither live nor attenuated viral vectors are used in their manufacturing. Additionally, the robust and rapid humoral and cell-mediated antiviral responses (over 90 percent) are absolutely astonishing when compared to vaccines such as seasonal flu vaccines, which have an efficacy of roughly 50 to 60 percent (Zhang et al., 2019). Last but not least, insertional mutagenesis is highly improbable since mRNA is not incorporated into the nucleus DNA material of immune cells; rather, it is gradually destroyed in the vesicles found in the cytoplasm the cell (Samaranayake et al., 2021).

**TABLE 1:** Advantages and disadvantages of commonly used COVID-19 vaccine platforms.

Vaccine type	Advantages	Disadvantages	Ongoing phase II/III trials
Live attenuated virus	Targeting and stimulation of robust immune response	Insufficient viral clearance in immunocompromised patients, the transition of the virus through feces to unvaccinated individuals, elevated risk of recombination between the vaccine strain and the wild infection	8 studies
Inactivated virus	High safety profile in the vaccinee	Epitope alteration during inactivation, needing high-tech facilities (biosafety level 3), incomplete inactivation, slow production compared to high demands for vaccines	76 studies
Virus-like particles	High cellular and humoral immune response due to the mimicking wild type of virus, no replication and person to person transition, ability to be loaded with immune-modulators	Less immunogenicity compared to other platforms	4 studies
Subunits	Safe enough after administration to vaccinees	Less immunogenicity compared to other platforms, need for adjuvant, stimulating mainly humoral immune response	22 studies
Viral vector	Feasible for high yield production, stimulating both immune responses	Ineffectiveness due to the prior infection with vector, interruption with future vaccine utilizing the same vector due to the memorial immunity against vector, increased chance of viral recombination	27 studies
DNA	targeting and boosting both humoral and cellular immune responses, quick and easy large-scale manufacturing and formulation methods, multivalency flexibility, final vaccine keeping at room temperature	Less immunogenicity compared to other platforms and need for booster, elevated chance of carcinogenicity due to the integration in chromosomes	18 studies
mRNA	Targeting both humoral and cellular immune responses, no integration within chromosomes	Expensive compared to other platforms, need for low temperature, unknown mechanism of degradation	81 studies

Despite the benefits listed above, the most significant disadvantage of these vaccines is their fragility. Since RNA is rapidly degraded, the formulations require ultra-cold chains (−70 C) for durability and stability. As a result, the widespread use of these vaccines in resource-poor jurisdictions is extremely dubious, as DNA vaccines are significantly superior in these areas (Because DNA is extremely stable, it can be retrieved even from prehistoric animal remains.) Furthermore, in this particular setting, mRNA vaccines have traditionally only been used for the treatment of cancer, not for the prevention of infections (Pardi et al., 2018). As a direct result, the potential adverse consequences that viral RNA vaccinations may have over the long term remain unclear. mRNA vaccines have the ability to modify quickly, for example, after the identification of SARS-CoV-2 variants, updated mRNA vaccines were announced in a short period of time, booster doses of mRNA vaccines were rapidly adapted to target emerging variants (Choi et al., 2021). Viral vector vaccines, however, need more time to update because they require the transfer of the new variant genetic material in the vector.

In a systematic review and meta-analysis in 2024, among the 284 evaluated articles, 11 were finally included in the analysis. The results showed that mRNA-based, inactivated vaccines and non-replicating viral vector-based vaccines provided significant protection compared to placebo. In this study, mRNA-based vaccines were more effective than other platforms (Beladiya et al., 2024). In another systematic review and meta-analysis in 2024 to assess vaccines in phase III trials, different types of vaccines did not show significant efficacy and all had an acceptable level. Also, BNT162b2 had the highest efficacy in preventing symptomatic SARS-CoV-2 infection in adults and the elderly (Wu et al., 2024). Another systematic review and meta-analysis in 2023 aimed at evaluating the efficacy and safety of mRNA COVID-19 in children aged 5–11 years showed that vaccination significantly reduced the risks of inflammation and hospitalization in these children (Watanabe et al., 2023). However, another meta-analysis found that primary vaccination with BNT162b2 and mRNA-1273 was likely to be effective in preventing SARS-CoV-2 infection and symptoms of COVID-19 in children aged 5–11 years before the onset of omicron (Piechotta et al., 2023). In the study by Zhi-Rong Yang et al., SARS-CoV-2 vaccines were more effective in controlling severe infections, however, their effectiveness decreased over time (Yang et al., 2023).

According to Kouhpayeh and Ansari’s systematic and meta-analysis, mRNA vaccines are associated with more side effects after immunization, the pool RRs of total adverse reactions for inactivated, mRNA, and vector vaccines were 1.46, 2.01, and 1.65, respectively (Kouhpayeh and Ansari, 2022). In the study of Farah Yasmin et al. also cardiovascular complication, Thrombosis, and thrombocytopenia have been reported after receiving mRNA vaccines (Adverse events following COVID‐19 mRNA vaccines (Yasmin et al., 2023).

## 9 Future perspective and conclusion

Despite the fact that the path to stopping the COVID-19 pandemic is still undetermined, vaccines have given the globe hope since studies indicate a considerable drop in the likelihood of COVID-19 infections and hospitalizations. On the other hand, some evidence suggests that vaccines lose some of their efficacy against illness over time. SARS-CoV-2 eradication is nearly impossible; therefore, the focus should be on minimizing COVID-19 severity and mortalities, with infection prevention as a future purpose. This may demand the development and implementation of vaccines based on novel strategies or the optimization of vaccines for novel SARS-CoV-2 variants. Despite this, it is imperative that the spread of SARS-CoV-2 be continuously monitored in every world region. COVID-19’s destructive impact has sparked tremendous vaccination and vaccine technology development in the battle against the pandemic. The consideration of current study was done on the literature which have been published until present, considering inclsion criteria for gathering and assessing the published researches. The obtained results demonstrated that most investigations have been done on mRNA- and inactivated virus-based vaccines with 81 studies and 76 studies, respectively. The shared advantages of both are being more effective on new strains and targeting all aspects of immune system. However, the drawbacks of former are being extremely expensive and need of further studies to find their degradation mechanisms. Those for the latter are need of high-tech facilities and incomplete inactivating process. Many COVID-19 vaccination programs were launched worldwide within a year of the disease’s outbreak, and more than 70 vaccines have advanced to clinical trials. Several of these have received the conditional license, and more are expected to do so in 2021. Several innovative technologies, such as mRNA vaccines and nonreplicating adenovirus vaccines, have rapidly come to prominence by winning the race for mass manufacturing and distribution and securing conditional permissions. This is in contrast to the majority of vaccines in development, which make use of conventional methodologies. These accomplishments are the result of many years’ worth of research, the timely dissemination of important information regarding the virus genome, an increase in collaboration between various research organizations, such as universities and pharmaceutical/biotech companies, increased support from the government, and, most importantly, the ceaseless efforts of vaccine researchers who have been working around the clock. The research’s success has been influenced by each of these elements. Now, COVID-19 mRNA vaccinations are essential in controlling the current epidemic’s progress. mRNA vaccines, in contrast to conventional vaccinations, may allow antigen design to be modified and even sequences from multiple varieties to be merged in order to adapt to new alterations in the viral genome. The future management and treatment of infectious illnesses as well as other issues will be made possible by the mRNA technology platform. Due to its advantages, including a quick development cycle, the lack of a cell culture need, and strong immunogenicity, the FDA has authorized an mRNA vaccine as the first COVID-19 vaccine to be produced globally.

The path from vaccine discovery to worldwide herd immunity against COVID-19 is fraught with policy problems requiring coordinated, global actions. Despite the significant accomplishment of bringing numerous vaccine candidates to market in less than a year, many obstacles remain in obtaining herd immunity at the national level and even more so at the global level. Decision-makers need to be aware of these issues and start thinking of solutions that may be adopted on a large scale. Only then will the world’s public health community be able to put a stop to one pandemic while also preparing for the next.
